# The Association of Heart Failure and Edema Events between Patients Initiating Sodium Zirconium Cyclosilicate or Patiromer

**DOI:** 10.34067/KID.0000000586

**Published:** 2024-09-20

**Authors:** Nihar R. Desai, Jennifer Kammerer, Jeffrey Budden, Abisola Olopoenia, Asa Tysseling, Alexandra Gordon

**Affiliations:** 1 Section of Cardiovascular Medicine, Department of Medicine, Yale School of Medicine, New Haven, Connecticut; 2 CSL Vifor, Redwood City, California; 3 Cerner Enviza, Malvern, Pennsylvania

**Keywords:** CKD, electrolytes, heart failure, hospitalization, renin-angiotensin system

## Abstract

**Key Points:**

- One previous study using claims data reported signals for higher hospitalizations for heart failures and severe edema in patients prescribed a potassium binder using sodium exchange.
- In this study, sodium zirconium cyclosilicate use was associated with increased risk of prespecified encounters of hospitalizations for heart failure and major edema encounters.
- Our findings highlight the need to weigh the benefits and risks of sodium zirconium cyclosilicate and patiromer in routine clinical practice.

**Background:**

Sodium zirconium cyclosilicate (SZC) and patiromer (PAT) are potassium binders that differ by exchange ion, sodium, and calcium, respectively. There are limited data on whether using sodium exchange could affect the risks of hospitalizations for heart failure (HHF) or severe edema in patients with hyperkalemia. The goal of this study was to assess the occurrence rates of prespecified major encounters potentially related to electrolyte-/fluid-related imbalances (including HHF, edema) among new users of PAT or SZC.

**Methods:**

Using Cerner Real World Data, we conducted a retrospective cohort study among adults (≥18 years) who were newly initiated on SZC or PAT between June 1, 2018, and December 31, 2021. Based on baseline demographic and clinical characteristics, one PAT initiator was propensity score matched with two SZC initiators. Primary outcomes were any HHF, primary HHF, major edema encounter, or death. Cox proportional hazard regression models were used to estimate the association between SZC or PAT use and each outcome in the overall population and subgroups with/without prior heart failure (HF).

**Results:**

The final cohort included 9929 PAT initiators matched to 19, 849 SZC initiators. The mean age was 66 years; about 50% had a history of CKD stages 3–5 and 34% a history of HF. Incidence rates were significantly higher in the SZC cohort when compared with the PAT cohort for all outcomes. Risks of HHF (any/primary) (adjusted hazard ratios [HRs], 1.373; 95% confidence interval [CI], 1.337 to 1.410), major edema encounter (HR, 1.330; 95% CI, 1.298 to 1.363), and death (HR, 1.287; 95% CI, 1.255 to 1.320) were also significantly higher in the SZC cohort compared with the PAT cohort (*P* < 0.05). These findings were consistent among subgroups with/without prior HF.

**Conclusions:**

SZC use (versus PAT) was associated with an increased risk of prespecified encounters that were potentially sodium-/fluid-related, including among patients with/without preexisting HF.

## Visual Abstract

**Figure:**
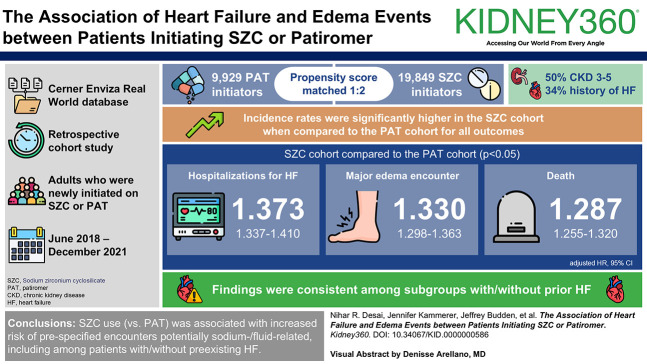

## Introduction

Hyperkalemia is a common complication in patients with CKD, heart failure (HF), and diabetes.^1–4^ Use of guideline recommended renin-angiotensin-aldosterone system (RAAS) inhibitors in these patients increases the risk of hyperkalemia.^4^ Current kidney disease guidelines recommend treating hyperkalemia with diet, diuretics, sodium bicarbonate, and/or potassium binders, as opposed to discontinuing renin-angiotensin aldosterone system inhibitors.^5^ Relative to the general population, patients with hyperkalemia have higher rates of health care resource utilization and increased overall health care costs.^6–9^

New potassium binders like patiromer (PAT) and sodium zirconium cyclosilicate (SZC) are approved to treat hyperkalemia by binding to and increasing fecal excretion of potassium.^10–13^ PAT is a nonabsorbed polymer and the only potassium binder that, by intentional design, uses a sodium-free exchange mechanism by exchanging potassium for calcium ions; by contrast, SZC is a nonabsorbed zirconium silicate that exchanges potassium for sodium and hydrogen ions.^13^ Both SZC and PAT effectively lower serum K^+^ concentrations in patients with hyperkalemia.^14^ Of note, the SZC label has both a warning/precaution about and adverse event mention of edema (4%–16% of patients), with neither included in the PAT label. The US Prescribing Information advises monitoring for signs of edema, especially in patients who need to limit their sodium intake or those at risk for fluid overload, such as those with HF or renal disease.^10^ The increased incidence of edema with SZC could be linked to sodium exchange, as each 5 g dose of SZC contains roughly 400 mg of sodium, which is equivalent to 20% of the World Health Organization recommended maximum daily sodium intake.^15^ The recommended starting dose in the US Prescribing Information for SZC in patients not on hemodialysis is 10 g three times a day until normokalemia is achieved, followed by 10 g maintenance dosing.

No direct head-to-head randomized studies comparing PAT and SZC have been completed. The majority of those completed have focused on changes in potassium levels as the primary outcome.^16–19^ Limited evidence is available on clinical (morbidity/mortality) outcomes or on safety signals potentially attributable to different exchange ions. In a recent study using single-payer claims data of Medicare Advantage and commercially insured patients, Zhuo *et al.* reported a significant increased risk of hospitalizations for HF (HHF) in patients without history of HF and severe edema among new users of SZC when compared with PAT users; other end points were consistently numerically in favor of PAT, but not statistically different.^20^ This study aimed to expand on these results using current electronic health record (EHR) data, which addresses some limitations associated with claims data, including biases associated with reimbursement coding, the influence of insurance coverage and type, and a lack of granular clinical and assessment data.

## Methods

### Study Design and Data Sources

This retrospective cohort study used the structured data from the Cerner Enviza Real World database. Cerner Enviza data are extracted from the EHRs of hospitals and clinics that have consented to such use and include data on pharmacy, clinical and microbiology laboratory, admission, and billing information. Cerner Enviza EHR data encompass de-identified data from over 100 million patients in the United States, including 642 million outpatient encounters, 43 million inpatient encounters, 101 million emergency encounters, 440 million condition encounters, 266 million medication encounters, and 298 million procedure encounters. In addition, the EHR data also contain information on patient demographics, including gender, birth sex, insurance type, marital status, race/ethnicity, and region.

### Study Population

The study population included all adults age ≥18 years who newly initiated PAT or SZC between June 1, 2018, and December 31, 2021 (prescription start as index date). As a proxy for continuous enrollment, individuals had to have had at least one health care encounter (in any setting) in the 180 days before the index date (preindex period) and in the 180 days after the index date (postindex period). Participants with prior use of SZC or PAT in the preindex period were excluded, as were those who switched from the index drug to the other within 3 days, to rule out changes due to formulary or access matters. Similar to Zhuo *et al.*, individuals with a history of kidney transplant or dialysis during the preindex period were excluded. Participants were followed from their index date until medication switch (if greater than 3 days), occurrence of prespecified events, discontinuation of medication (based on a gap of 30 days), death, or end of postindex period, whichever occurred first. To the extent possible, this study used methodology and examined outcomes mirroring the Zhuo *et al.* design, which was conducted independent of any declared pharmaceutical industry involvement. This study was reviewed and approved by the Pearl Institutional Review Board (Indianapolis, IN; 19-KANT-204) (Supplemental Table 1).

### Study Variables

The primary outcomes assessed in the postindex period included HHF, edema-related hospitalization or endothelial dysfunction encounter, or mortality; HHF and edema were captured based on diagnostic (International Classification of Diseases and Systemized Nomenclature of Medicine [SNOMED]) codes. HHF was further categorized into any HHF (on the basis of any inpatient visits with an associated HF code) and primary HHF (on the basis of any inpatient visits with a final discharge diagnosis of HF).

Demographic variables, including age, race/ethnicity, sex, region, insurance type, and marital status, were captured in the preindex period. Clinical characteristics, including prespecified comorbidities—such as CKD, diabetic nephropathy, and HF—body mass index, and prespecified prescribed medications, were assessed in the preindex period. Comorbidities and prescription medications were identified on the basis of diagnostic and national drug codes, respectively.

### Statistical Analysis

Demographic and clinical characteristics of patients in the PAT population were compared with those in the SZC population using descriptive and bivariate analyses. Frequencies and percentages were reported for categorical variables, while means and SD were reported for continuous variables. Propensity scores, estimated *via* logistic regression, were used to match two SZC users to one PAT user. Covariates in propensity score matching (PSM) were selected on the basis of clinical relevance to HF and statistical significance from the unmatched bivariate results; this included age; sex; ethnicity; history of any HF, stage 3–5 CKD, and diabetic nephropathy; and use of sodium polystyrene sulfonate (SPS), RAAS inhibitors, mineralocorticoid receptor antagonists, *β*-blockers, loop diuretics, and sodium-glucose cotransporter-2 inhibitors. Balance was assessed by calculating standardized mean differences (SMD) between the matched cohorts, and an SMD of 0.10 was considered the threshold for assessing balance. Bivariate analyses of predetermined outcomes were conducted on the matched cohorts: continuous variables were compared with *t* tests and categorical variables were compared with chi-squared tests. *P* values < 0.05 were considered statistically significant.

Cox proportional hazard regression models were used to estimate the association between SZC or PAT use and each outcome (any HHF, primary HHF, severe edema, and death) separately. Analyses were completed in the overall matched population and within subgroups of patients with and without prior HF. Incidence rates (IRs), rate differences (RDs), and unadjusted and adjusted hazard ratios (HRs) with corresponding 95% confidence interval (CI) were reported for each analysis. All analyses were performed using SAS v.9.4 or higher or R v.4.0.2 or higher version.

## Results

### Patient Characteristics

**Figure 1:**
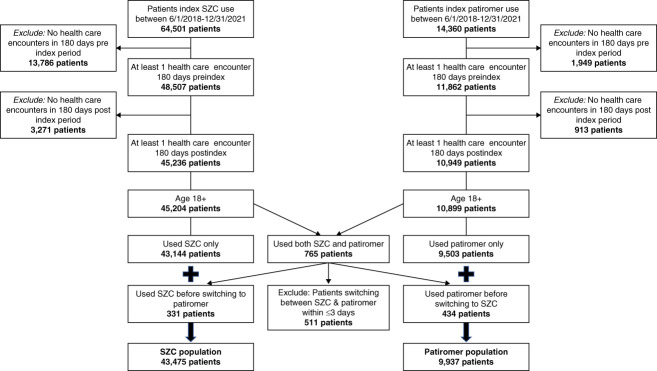
**Study cohort.** SZC, sodium zirconium cyclosilicate.

The final study population included 53,412 patients, of whom 9937 initiated treatment with PAT and 43,475 initiated therapy with SZC (Figure 1). Of them, 9929 PAT initiators (99.9%) were matched to 19,849 SZC initiators (45.7%). The full baseline demographic and clinical characteristics of study participants before PSM are shown in Supplemental Table 2. Before matching, a majority of sociodemographic and clinical characteristics differed significantly between the PAT and SZC cohorts (*P* < 0.05) (Table 1 and Supplemental Table 2). A majority of patients were ≥65 years (53.4%), female (57.8%), White (59.9%), of non-Hispanic descent (73.7%), unmarried (55.1%), resided in the Western region of the United States (40.8%), and had Medicare insurance (49.7%).

**Table 1:** Baseline sociodemographic and clinical characteristics

Characteristic	Unmatched Treatment Cohorts			Matched Treatment Cohorts^a^			
	SZC (*n*=43,475)	PAT (*n*=9937)	*P* Value	SZC (*n*=19,849)	PAT (*n*=9929)	*P* Value	SMD
Age in years, mean (SD)	*n*=43,436	*n*=9930	<0.001^b^	66.0 (13.8)	65.8 (14.3)	0.196^b^	0.016
	64.2 (14.8)	65.8 (14.3)					
**Sex, *No.* (%)**							
Female	25,163 (57.9)	5689 (57.3)	0.280^b^	8312 (41.9)	4229 (42.6)	0.441^b^	0.016
**Ethnicity, *No.* (%)**							
Hispanic	10,975 (25.2)	2210 (22.2)	<0.001^b^	4277 (21.5)	2209 (22.2)	0.178^b^	0.023
**Comorbidities, *No.* (%)**							
Any HF	16,003 (36.8)	3362 (33.8)	<0.001^b^	6615 (33.3)	3360 (33.8)	0.376^b^	0.011
CKD (stage 3–5)	20,570 (47.3)	4932 (49.6)	<0.001^b^	9830 (49.5)	4929 (49.6)	0.847^b^	0.002
Diabetic nephropathy	16,354 (37.6)	4197 (42.2)	<0.001^b^	8230 (41.5)	4195 (42.2)	0.194^b^	0.016
**Concomitant medications, *No.* (%)**							
ACE inhibitor (any combinations)	12,528 (28.8)	2655 (26.7)	<0.001^b^	8677 (43.7)^b^	4348 (43.8)^b^	0.901^b^	0.002
ARBs (any combinations)	7667 (17.6)	1751 (17.6)	0.973^b^				
Sacubitril/valsartan	1050 (2.4)	276 (2.8)	0.036^b^				
MRA (any combinations)	5281 (12.1)	978 (9.8)	<0.001^b^	1837 (9.3)	977 (9.8)	0.104^b^	0.020
Any *β*-blockers (any combinations)	28,361 (65.2)	6593 (66.3)	0.035^b^	13,285 (66.9)	6587 (66.3)	0.309^b^	0.012
Loop diuretics (any combinations)	25,229 (58.0)	5497 (55.3)	<0.001^b^	10,979 (55.3)	5495 (55.3)	0.960^b^	0.001
SGLT-2 inhibitors (any combinations)	1068 (2.5)	167 (1.7)	<0.001^b^	283 (1.4)	167 (1.7)	0.088^b^	0.021
SPS	7094 (16.3)	2915 (29.3)	<0.001^b^	5700 (28.7)	2913 (29.3)	0.265^b^	0.014

*P* value > 0.05 and SMD <0.10 indicate no significant differences between groups. ACE, angiotensin-converting enzyme; ARB, angiotensin receptor blockers; HF, heart failure; MRA, mineralocorticoid receptor antagonist; PAT, patiromer; SGLT-2, sodium-glucose cotransporter-2; SMD, standardized mean difference; SPS, sodium polystyrene sulfonate; SZC, sodium zirconium cyclosilicate.

a All SMD were <0.10 for all variables in the matched cohort.

b Renin-angiotensin-aldosterone system inhibitors (ACE inhibitor, ARB, and/or any combination products with such including sacubitril/valsartan). This group was not broken down into the subcomponents in the matched cohort.

c Renin-angiotensin-aldosterone system inhibitors (ACE inhibitor, ARB, and/or any combination products with such, including sacubitril/valsartan).

After matching, all patient characteristics were well balanced between both cohorts (all, *P* ≥ 0.05; SMDs ≤0.10). Approximately one-third of patients had a history of HF (PAT: 33.8% versus SZC: 33.3%), about half had comorbid CKD (stage 3–5) (each, 50.0%), and over two in five patients had a diagnosis of diabetic nephropathy (each, 42.0%). The most common medications concurrently prescribed were *β*-blockers (66.3%, PAT versus 66.9%, SZC), followed by loop diuretics (both, 55.3%), RAAS inhibitors (both, 44.0%), SPS (both 29.0%), mineralocorticoid receptor antagonists (9.8% versus 9.3%), and sodium-glucose cotransporter-2 inhibitors (1.7% versus 1.4%; all *P* > 0.05; Table 1).

### Primary Outcomes: HF, Edema, and Mortality

#### Overall Matched Population

Adjusted comparisons reflecting the incidence and risk of outcomes in matched treatment cohorts (*n*=9,923, PAT and *n*=19,840, SZC) are presented in Table 2. The IR for all outcomes examined were significantly higher in the SZC cohort when compared with the PAT cohort (HHF [any]: IR—93 per 1000 versus 71 per 1000 person-months; primary HHF: IR—74 per 1000 person-months versus 65 per 1000 person-months; death: IR—54 per 1000 person-months versus 26 per 1000 person-months; severe edema: IR—15 per 1000 person-months versus 9 per 1000 person-months).

**Table 2:** Number of events, IRs, RDs, and HRs for study outcomes in 1:2 PSM matched treatment cohorts: overall population analysis

Outcome	SZC (*n*=19,840), *No.* (IR)	PAT (*n*=9923), *No.* (IR)	SZC versus PAT		
			RD (95% CI)	Crude HR (95% CI)	Adjusted HR (95% CI)
Hospitalization for HF-any^a^	2977 (0.093)	1590 (0.071)	0.022 (0.017 to 0.026)^b^	1.40 (1.363 to 1.430)^b^	1.37 (1.337 to 1.410)^b^
Hospitalization for HF-(discharge diagnosis only)^c^	2433 (0.074)	1474 (0.065)	0.009 (0.004 to 0.013)^b^	1.40 (1.365 to 1.431)^b^	1.37 (1.337 to 1.409)^b^
Death^d^	1954 (0.054)	627 (0.026)	0.028 (0.025 to 0.032)^b^	1.31 (1.285 to 1.344)^b^	1.29 (1.255 to 1.320)^b^
Severe edema^e^	522 (0.015)	221 (0.009)	0.006 (0.004 to 0.007)^b^	1.35 (1.322 to 1.382)^b^	1.33 (1.298 to 1.363)^b^

IRs may be underestimated because the last day of the month was used as a proxy for the date of death. CI, confidence interval; HF, heart failure; HR, hazard ratio; IR, incidence rate; PAT, patiromer; PSM, propensity score matching; RD, rate difference; SZC, sodium zirconium cyclosilicate.

a SZC person-months: 32,044.43; patiromer person-months: 22,321.86.

b Significant findings.

c SZC person-months: 32,878.87; patiromer person-months: 22,538.02.

d SZC person-months: 36,210.66; patiromer person-months: 24,539.84.

e SZC person-months: 35,504.61; patiromer person-months: 24,225.25.

On the basis of differences in IRs, and compared with the PAT cohort, those in the SZC cohort had 22 more first HHF-any (RD, 0.022; 95% CI, 0.017 to 0.026) and nine more HHF-primary (RD, 0.009; 95% CI, 0.004 to 0.013) encounters per 1000 person-months. Furthermore, compared with PAT initiators, those treated with SZC experienced 28 more all-cause deaths (RD, 0.028; 95% CI, 0.025 to 0.032) and six more severe edema events (RD, 0.006; 95% CI, 0.004 to 0.007) per 1000 person-months. Relative to the PAT cohort, SZC users had 37.3% higher risk of HHF-any (HR, 1.373; 95% CI, 1.337 to 1.410) and HHF-primary (HR, 1.373; 95% CI, 1.337 to 1.409), 28.7% higher risk of all-cause death (HR, 1.287; 95% CI, 1.255 to 1.320), and 33.0% higher risk of severe edema (HR, 1.330; 95% CI, 1.298 to 1.363; Table 2). These findings were all statistically significant.

#### Subgroup Analysis

Subgroup analysis of the incidence and risk of health events in matched treatment cohorts of patients with no history of HF (*n*=13,229, SZC and *n*=6,564, PAT) and those with a history of HF (*n*=6,611, SZC and *n*=3,359, PAT) suggested a similar pattern, as was noted in the main analyses (Table 3).

**Table 3:** Number of events, IRs, RDs, and HRs for study outcomes in 1:2 propensity score matching matched treatment cohorts: Subgroup analysis

Outcome	Patients with No History of HF					Patients with Prior HF				
	SZC (*n*=13,229), *No.* (IR)	PAT (*n*=6564), *No.* (IR)	SZC versus PAT			SZC (*n*=6611), *No.* (IR)	PAT (*n*=3359), *No.* (IR)	SZC versus PAT		
			RD (95% CI)	Crude HR (95% CI)	Adjusted HR (95% CI)			RD (95% CI)	Crude HR (95% CI)	Adjusted HR (95% CI)
HHF-any^a^	718 (0.031)	362 (0.022)	0.009 (0.006 to 0.012)^b^	1.42 (1.381 to 1.460)^b^	1.39 (1.348 to 1.433)^b^	2259 (0.262)	1228 (0.217)	0.045 (0.028 to 0.061)^b^	1.34 (1.277 to 1.403)^b^	1.33 (1.259 to 1.398)^b^
HHF-primary^c^	594 (0.025)	332 (0.020)	0.005 (0.002 to 0.008)^b^	1.42 (1.384 to 1.464)^b^	1.39 (1.350 to 1.436)^b^	1839 (0.198)	1142 (0.197)	0.001 (-0.014 to 0.015)	1.34 (1.278 to 1.400)^b^	1.32 (1.257 to 1.392)^b^
Death^d^	1167 (0.048)	317 (0.018)	0.029 (0.026 to 0.033)^b^	1.36 (1.327 to 1.403)^b^	1.33 (1.290 to 1.372)^b^	787 (0.067)	310 (0.042)	0.024 (0.018 to 0.031)^b^	1.21 (1.165 to 1.260)^b^	1.20 (1.143 to 1.248)^b^
Severe edema^e^	294 (0.012)	105 (0.006)	0.006 (0.004 to 0.008)^b^	1.41 (1.372 to 1.449)^b^	1.38 (1.338 to 1.422)^b^	228 (0.020)	116 (0.016)	0.004 (0.000 to 0.008)	1.23 (1.188 to 1.281)^b^	1.23 (1.177 to 1.282)^b^

IRs may be underestimated because the last day of the month was used as a proxy for the date of death. CI, confidence interval; HF, heart failure; HHF, hospitalizations for heart failure; HR, hazard ratio; IR, incidence rate; PAT, patiromer; RD, rate difference; SZC, sodium zirconium cyclosilicate.

a Patients with no history of HF—SZC person-months: 23,423.36; patiromer person-months: 16,674.62. Patients with prior HF—SZC person-months: 8621.07; patiromer person-months 5647.24.

b Significant findings.

c Patients with no history of HF—SZC person-months: 23,590.81; patiromer person-months: 16,752.27. Patients with prior HF—SZC person-months: 9288.69; patiromer person-months: 5785.76.

d Patients with no history of HF—SZC person-months: 24,421.90; patiromer person-months: 17,233.15. Patients with prior HF—SZC person-months: 11,489.93; patiromer person-months: 7171.86.

e Patients with no history of HF—SZC person-months: 24,014.68; patiromer person-months: 17,053.39. Patients with prior HF—SZC person-months: 11,489.93; patiromer person-months: 7171.86.

### Patients with No History of HF during Preindex Period

There were nine more HHF-any encounters in the SZC cohort compared with PAT users (RD, 0.009; 95% CI, 0.006 to 0.012) and five more HHF-primary encounters (RD, 0.005; 95% CI, 0.002 to 0.008) per 1000 person-months. In addition, compared with PAT initiators, those prescribed SZC experienced 29 more deaths (RD, 0.029; 95% CI, 0.026 to 0.033) and six more severe edema events (RD, 0.006; 95% CI, 0.004 to 0.008) per 1000 person-months. Adjusted comparisons showed that among patients with no history of HF, SZC users had 39% higher risk of any HHF (HR, 1.390; 95% CI, 1.348 to 1.433), 39.2% higher risk of primary HHF (HR, 1.392; 95% CI, 1.350 to 1.436), 33.1% higher risk of death (HR, 1.331; 95% CI, 1.290 to 1.372), and 37.9% higher risk of severe edema (HR, 1.379; 95% CI, 1.338 to 1.422; Table 3) when compared with PAT users, and these findings were statistically significant.

### Patients with Prior HF during Preindex Period

Among patients with prior HF, there were 45 more hospitalizations for any HHF (RD, 0.045; 95% CI, 0.028 to 0.061) per 1000 person-months and 24 more deaths (RD, 0.024; 95% CI, 0.018 to 0.031) per 1000 person-months. Adjusted comparisons showed that among patients with prior HF, SZC users had 32.6% higher risk of HHF-any (HR, 1.326; 95% CI, 1.259 to 1.398), 32.3% higher risk of HHF-primary (HR, 1.323; 95% CI, 1.257 to 1.392), 19.5% higher risk of all-cause death (HR, 1.195; 95% CI, 1.143 to 1.248), and 22.8% higher risk of severe edema (HR, 1.228; 95% CI, 1.177 to 1.282; Table 3) when compared with PAT users, all statistically significant.

## Discussion

In this retrospective cohort study, a majority of individuals newly initiating PAT or SZC were older than 65 years, more than a third were previously diagnosed with HF, about half had a history of CKD stage 3–5, approximately one-third had a history of HF, and about half were prescribed RAAS inhibitors. The study demonstrated that relative to PAT, initiation of SZC was associated with an increased risk of HHF, mortality, and severe edema in a large and representative population from real-world settings. These findings were consistent across patients with and without HF history.

Hyperkalemia is common among patients with CKD, especially among those who are prescribed RAAS inhibitor medications. New potassium-lowering agents, SZC, and PAT have proven their efficacy in large clinical trials.^17–22^ PAT uses sodium-free exchange of calcium for potassium, whereas SZC exchanges sodium and hydrogen for potassium.^13^ Increased sodium may result in fluid retention, edema, and potentially increased risk of HF. The HARMONIZE trial first reported a dose-dependent increase in edema after 1 month of treatment (2% placebo, 2% 5 g SZC, 6% 10 g SZC, and 14% 15 g SZC).^18^ The recent PRIORITIZE HF trial reported numerically more patients with worsening HF in the SZC group (11 patients [12.1%]), compared with the placebo group (five patients [5.6%]) over 3 months of study.^23^ In the 1-year ZS-005 study evaluating the efficacy and safety of SZC for the long-term treatment of patients with hyperkalemia, edema (evaluated by standardized Medical Dictionary for Regulatory Activities query [SMQ edema] for hemodynamic edema, effusions, and fluid overload) was reported in 15% of patients and was higher in patients with CKD or HF.^24^ For context, peripheral edema was reported in approximately 4% of patients with diabetic kidney disease. The higher frequency of severe edema events (33%) observed among new SZC users (versus PAT users) in this study is in line with the SZC US PI^15^ and existing literature, including Zhuo *et al.,* on which the design of this study is largely based.^20,22,25^

This study also noted that new users of SZC had 37% higher risk of HF-related hospitalizations and 29% higher risk of death, when compared with PAT users. The directionality of associations in this study is consistent with those reported by Zhuo *et al.*^20^ —22% increased risk of any HHF, 15% increased risk of primary HHF, and 16% increased risk of death among SZC users when compared with PAT users. However, where not all associations in Zhuo *et al.* were statistically significant, all results in this study in both the overall and subgroups with and without prior HF showed consistently significant results. In subgroup comparisons, our study noted an increased likelihood of HHF, death, and severe edema events among SZC users across all populations, regardless of their history of HF. Importantly, the risk of all events was significantly higher among patients with no HF history; specifically, among patients with no HF history, SZC initiation was associated with a 39% increased risk of HHF (versus 32% for prior HF), a 33.1% increased risk of mortality (versus 19.5% for prior HF), and a 37.9% increased risk of edema (versus 22.8% for prior HF) when compared with PAT use. This finding is consistent with Zhuo and colleagues, which noted that among patients with no history of HF, SZC use was associated with a 58% higher risk of HHF.

Differences in the noted magnitude and direction of other associations between this study and the study by Zhuo *et al.* may be related to differences in between-study populations. Zhuo and colleagues captured an older population, with a greater proportion of CKD stage 3–5 and who were primarily insured by a single payer and by Medicare Advantage. On the other hand, this study included a much larger study population covered by multiple payers and insurance types, including commercial, Medicare Advantage, Medicaid Managed care, and others, and more diverse in terms of race and ethnicity. Although our methodological approach for this study was designed to closely mirror the approach used in the study by Zhuo, there are some important differences. In the study by Zhuo, one SZC user was matched to three PAT users, with the relevant period for assessing medication use between May 18, 2018, and September 30, 2020. However, there were more SZC users in our study population, and one PAT user was matched to two SZC users. This possibly reflects market trend with more adoption of SZC, as we extended our study period beyond 2020 until December 31, 2021. In addition, to address potential confounding, a comprehensive list of about 80 variables were included in the PSM criteria used in the study by Zhuo. In our study, however, to minimize collinearity, variables were selected in the PSM on the basis of statistical and clinical relevance.

Finally, in the study by Zhuo, individuals were followed from their index date until the occurrence of the event, switch/addition of other medication, discontinuation of index therapy, end of continuous enrollment, or end of the study period. Because our study was based on EHR data, we do not have access to continuous enrollment information, and follow-up was limited to 6 months postindex or until any of the other criteria previously listed in the study by Zhuo. However, we do not believe this affected our study findings because median follow-up was similar in both studies (median follow-up in the study by Zhuo: 58 days; median follow-up in our study: 35–48 days). Furthermore, evidence suggests that the majority of SZC users have <3 months of use.^26^ Despite some differences, our findings, along with those reported by Zhuo *et al.*, suggest that treatment with SZC may be associated with higher risks of HF hospitalizations and severe edema in a subset of patients. In addition, although hyperkalemia was not included in the PSM for this analysis, laboratory data were assessed in this study—mean (SD) potassium levels were similar across treatment groups (SZC K: 5.5 [0.6] and PAT K: 5.4 [0.7]); as such, we do not believe this finding was due to potassium levels confounding the noted association.

This study has several notable strengths that enhance its overall robustness and relevance. To our knowledge, this is among the first studies to examine the association between events potentially related to sodium/fluid imbalance and SZC use by using EHR data. This strategic choice that avoids certain limitations often associated with reliance on reimbursement data. Furthermore, the data source for this study represents a diverse range of payers, insurance types, formulary and benefit designs, and health care systems, ensuring a more generalizable representation of the population. This study included a large study population of incident SZC and PAT users, thus not only enhancing the study's external validity, but also limiting the impact of prevalent user bias on the noted associations. This study also incorporated SNOMED codes to define outcomes, allowing for a better capture of events.

This study has a few limitations. First, there is a potential for selection bias because this study only captured individuals who sought care in a health care setting. Still, this Cerner Enviza Real World database captures all EHR data across all venues of care from multiple health systems; as such, health care encounters are less likely to be missed. The study did not compare either modern binder to SPS, which has a more intermittent real-world use pattern, making such comparisons difficult. It also did not directly assess duration of drug exposure, but follow-up time was very similar to Zhuo *et al.* (median, 58 days), and a body of claims-based evidence suggests the follow-up time closely mirrors duration of use for a majority of patients.^26–28^ This study was also not designed to compare efficacy between the products. In addition, there is a possibility for misclassification of outcomes; this was minimized by using a combination of diagnostic and SNOMED codes for the classification of outcomes. This study relies on prescribed medication data, which may not fully represent patients' actual medication usage. Death was assessed on the basis of EHR data; as a result, any deaths which occurred outside of the network would not be captured. Finally, despite the use of PSM, there remains the potential for residual confounding.

We noted that incident SZC use was associated with an increased risk of experiencing HF-related hospitalizations, severe edema events, and all-cause mortality, regardless of history of HF. This study provides valuable insights into the safety of SZC and PAT therapies for the management of hyperkalemia and suggests that the benefits and risks of these therapies need to be carefully considered in real-world clinical practice.

## Supplementary Material

## Data Availability

Data cannot be shared. Cerner Enviza Real World database is a commercial dataset. Access to the dataset used in this present paper must be requested to Oracle Life Sciences. Data access conditions will be detailed by the responsible team.

## Supplemental Material

Supplemental Table 1. NDC and Multum codes for patiromer and SZC.

Supplemental Table 2. Baseline sociodemographic and clinical characteristics.
