# Supplementary material for: The Association of Heart Failure and Edema Events between Patients Initiating Sodium Zirconium Cyclosilicate or Patiromer
Source: Kidney360. 2024 Sep 20;5(12):1835–43. doi: 10.34067/KID.0000000586 (PMC11687973; doi:10.34067/KID.0000000586)
Supplement: Supplementary file 1 [file kidney360-5-1835-s001.pdf]

ASN American Society of Nephrology | JASN Journal of the American Society of Nephrology | KIDNEY360 Accessing Our World From Every Angle | CJASN Clinical Journal of the American Society of Nephrology

ASN Journal Disclosure Form

As per ASN journal policy, I have disclosed any financial relationships or commitments I have held in the past 36 months as included below. I have listed my Current Employer below to indicate there is a relationship requiring disclosure. If no relationship exists, my Current Employer is not listed.

J. Budden reports the following:
Employer: Employment - CSL Vifor; and Ownership Interest: CSL Vifor.

I understand that the information above will be published within the journal article, if accepted, and that failure to comply and/or to accurately and completely report the potential financial conflicts of interest could lead to the following: 1) Prior to publication, article rejection, or 2) Post-publication, sanctions ranging from, but not limited to, issuing a correction, reporting the inaccurate information to the authors' institution, banning authors from submitting work to ASN journals for varying lengths of time, and/or retraction of the published work.

Name: Jeffrey Budden
Manuscript ID: K360-2024-000223R1
Manuscript Title: The Association of Heart Failure and Edema events between Patients Initiating SZC or Patiromer
Date of Completion: July 1, 2024
Disclosure Updated Date: July 1, 2024

ASN American Society of Nephrology | JASN Journal of the American Society of Nephrology | KIDNEY360 Accessing Our World From Every Angle | CJASN Clinical Journal of the American Society of Nephrology

ASN Journal Disclosure Form

As per ASN journal policy, I have disclosed any financial relationships or commitments I have held in the past 36 months as included below. I have listed my Current Employer below to indicate there is a relationship requiring disclosure. If no relationship exists, my Current Employer is not listed.

N. Desai reports the following:
Employer: Yale School of Medicine; Consultancy: Amgen, Astra Zeneca, Bayer, Bristol Myers Squibb, Boehringer Ingelheim, CSL Behring, Cytokinetics, Merck, Novartis, SC Pharmaceuticals, Verve Therapeutics, Vifor Pharmaceuticals.; Research Funding: Amgen, Astra Zeneca, Boehringer Ingelheim, Cytokinetics, Vifor; and Honoraria: Amgen, Bayer, Boehringer Ingelheim, CSL Behring, Cytokinetics, Novartis, Medicines Company, SC Pharmaceuticals, Verve Therapeutics.

I understand that the information above will be published within the journal article, if accepted, and that failure to comply and/or to accurately and completely report the potential financial conflicts of interest could lead to the following: 1) Prior to publication, article rejection, or 2) Post-publication, sanctions ranging from, but not limited to, issuing a correction, reporting the inaccurate information to the authors' institution, banning authors from submitting work to ASN journals for varying lengths of time, and/or retraction of the published work.

Name: Nihar Desai
Manuscript ID: K360-2024-000223R1
Manuscript Title: "The Association of Heart Failure and Edema events between Patients Initiating SZC or Patiromer"
Date of Completion: August 20, 2024
Disclosure Updated Date: August 16, 2024

ASN American Society of Nephrology
JASN Journal of the American Society of Nephrology
KIDNEY360 Accessing Our World From Every Angle
CJASN Clinical Journal of the American Society of Nephrology

ASN Journal Disclosure Form

As per ASN journal policy, I have disclosed any financial relationships or commitments I have held in the past 36 months as included below. I have listed my Current Employer below to indicate there is a relationship requiring disclosure. If no relationship exists, my Current Employer is not listed.

A. Gordon reports the following:
Employer: Oracle

I understand that the information above will be published within the journal article, if accepted, and that failure to comply and/or to accurately and completely report the potential financial conflicts of interest could lead to the following: 1) Prior to publication, article rejection, or 2) Post-publication, sanctions ranging from, but not limited to, issuing a correction, reporting the inaccurate information to the authors' institution, banning authors from submitting work to ASN journals for varying lengths of time, and/or retraction of the published work.

Name: Alexandra C Gordon
Manuscript ID: K360-2024-000223R1
Manuscript Title: The Association of Heart Failure and Edema events between Patients Initiating SZC or Patiromer
Date of Completion: July 1, 2024
Disclosure Updated Date: July 1, 2024

ASN Journal Disclosure Form

As per ASN journal policy, I have disclosed any financial relationships or commitments I have held in the past 36 months as included below. I have listed my Current Employer below to indicate there is a relationship requiring disclosure. If no relationship exists, my Current Employer is not listed.

J. Kammerer reports the following:
Employer: CSL Vifor (end 12/2023); Consultancy: SeaStar Medical, Inc; Ownership Interest: SeaStar Medical, Inc; CSL Vifor; Research Funding: SeaStar Medical, Inc; CSL Vifor; and Other Interests or Relationships: Medical Reserve Corps of Colorado.

I understand that the information above will be published within the journal article, if accepted, and that failure to comply and/or to accurately and completely report the potential financial conflicts of interest could lead to the following: 1) Prior to publication, article rejection, or 2) Post-publication, sanctions ranging from, but not limited to, issuing a correction, reporting the inaccurate information to the authors' institution, banning authors from submitting work to ASN journals for varying lengths of time, and/or retraction of the published work.

Name: Jennifer A. Kammerer
Manuscript ID: K360-2023-000806R2
Manuscript Title: Heart Failure and Edema Costs in Patiromer and SZC Users
Date of Completion: May 22, 2024
Disclosure Updated Date: May 21, 2024

ASN American Society of Nephrology · JASN Journal of the American Society of Nephrology · KIDNEY360 Accessing Our World From Every Angle · CJASN Clinical Journal of the American Society of Nephrology

ASN Journal Disclosure Form

As per ASN journal policy, I have disclosed any financial relationships or commitments I have held in the past 36 months as included below. I have listed my Current Employer below to indicate there is a relationship requiring disclosure. If no relationship exists, my Current Employer is not listed.

A. Olopoenia reports the following:
Employer: Oracle; Consultancy: Oracle; Ownership Interest: Oracle; and Research Funding: Pfizer inc.; Vifor; Boehringer Ingelheim; CSL Behring; Amgen; Otsuka.

I understand that the information above will be published within the journal article, if accepted, and that failure to comply and/or to accurately and completely report the potential financial conflicts of interest could lead to the following: 1) Prior to publication, article rejection, or 2) Post-publication, sanctions ranging from, but not limited to, issuing a correction, reporting the inaccurate information to the authors' institution, banning authors from submitting work to ASN journals for varying lengths of time, and/or retraction of the published work.

Name: Abisola Olopoenia
Manuscript ID: K360-2024-000223R1
Manuscript Title: The Association of Heart Failure and Edema events between Patients Initiating SZC or Patiromer
Date of Completion: July 1, 2024
Disclosure Updated Date: July 1, 2024

ASN American Society of Nephrology
JASN Journal of the American Society of Nephrology
KIDNEY360 Accessing Our World From Every Angle
CJASN Clinical Journal of the American Society of Nephrology

ASN Journal Disclosure Form

As per ASN journal policy, I have disclosed any financial relationships or commitments I have held in the past 36 months as included below. I have listed my Current Employer below to indicate there is a relationship requiring disclosure. If no relationship exists, my Current Employer is not listed.

A. Tysseling reports the following:
Employer: Oracle Cerner

I understand that the information above will be published within the journal article, if accepted, and that failure to comply and/or to accurately and completely report the potential financial conflicts of interest could lead to the following: 1) Prior to publication, article rejection, or 2) Post-publication, sanctions ranging from, but not limited to, issuing a correction, reporting the inaccurate information to the authors' institution, banning authors from submitting work to ASN journals for varying lengths of time, and/or retraction of the published work.

Name: Asa C. Tysseling
Manuscript ID: K360-2024-000223R1
Manuscript Title: The Association of Heart Failure and Edema events between Patients Initiating SZC or Patiromer
Date of Completion: September 3, 2024
Disclosure Updated Date: September 3, 2024
