# Supplementary material for: The Association of Heart Failure and Edema Events between Patients Initiating Sodium Zirconium Cyclosilicate or Patiromer
Source: Kidney360. 2024 Sep 20;5(12):1835–43. doi: 10.34067/KID.0000000586 (PMC11687973; doi:10.34067/KID.0000000586)
Supplement: Supplementary file 2 [file kidney360-5-1835-s002.pdf]

Table of Contents

Table 1. NDC and Multum codes for patiromer and SZC* 2
Table 2. Baseline sociodemographic and clinical characteristics 3

Table 1. NDC and Multum codes for patiromer and SZC*

	Code Type	Code	Description
**Patiromer**	Multum	d08388	Patiromer
	NDC	53436008401	Patiromer 8.4 g oral packet
	NDC	53436008404	Patiromer 8.4 g oral packet
	NDC	53436008430	Patiromer 8.4 g oral packet
	NDC	53436008491	Patiromer 8.4 g oral powder
	NDC	53436008492	Patiromer 8.4 g oral powder
	NDC	53436016801	Patiromer 16.8 g oral packet
	NDC	53436016830	Patiromer 16.8 g oral packet
	NDC	53436025201	Patiromer 25.2 g oral packet
	NDC	53436025230	Patiromer 25.2 g oral packet
**SZC**	Multum	d08797	SZC
	NDC	310110501	SZC 5 g powder packet
	NDC	310110530	SZC 5 g powder packet
	NDC	310110539	SZC 5 g powder packet
	NDC	310111001	SZC 10 g powder packet
	NDC	310111030	SZC 10 g powder packet
	NDC	310111039	SZC 10 g powder packet
	NDC	310111091	SZC 10000 mg powder for oral suspension
	NDC	310111094	SZC 10000 mg powder for oral suspension
	NDC	310111098	SZC 10000 mg powder for oral suspension

*Medication records were not limited to only NDC and Multum codes. All medication coding systems in CRWD were leveraged.

CRWD, Cerner Enviza EHR database; g, gram; mg, milligrams; NDC, National Drug Code; SZC, sodium zirconium cyclosilicate.

Table 2. Baseline sociodemographic and clinical characteristics

Characteristics	Unmatched treatment cohorts			
	**Total (N=53,412)**	**Patiromer (N=9,937)**	**SZC (N=43,475)**	***P*-value**
Age in years, mean (SD)	**n=53,366**	**n=9,930**	**n=43,436**	***<0.001***
	64.5 (14.7)	65.8 (14.3)	64.2 (14.8)	
Age in years, n (%)				
18-34	2,134 (4.0)	329 (3.3)	1,805 (4.2)	
35-44	3,308 (6.2)	491 (4.9)	2,817 (6.5)	
45-64	19,418 (36.4)	3,411 (34.3)	16,007 (36.8)	
65+	28,506 (53.4)	5,699 (57.4)	22,807 (52.5)	
Missing	46 (0.1)	7 (0.1)	39 (0.1)	
Sex, n (%)				
Male	22,503 (42.1)	4,234 (42.6)	18,269 (42.0)	*0.280*
Female	30,852 (57.8)	5,689 (57.3)	25,163 (57.9)	
Unknown/other	57 (0.1)	14 (0.1)	43 (0.1)	
Race, n (%)				
White	31,979 (59.9)	6,147 (61.9)	25,832 (59.4)	***<0.001***
Black or African-American	10,021 (18.8)	1,796 (18.1)	8,225 (18.9)	
Asian/American Indian/Alaskan Native/Native Hawaiian/other Pacific Islander	2,480 (4.6)	556 (5.6)	1,924 (4.4)	
Unknown[a]	8,932 (16.7)	1,438 (14.5)	7,494 (17.2)	
Ethnicity, n (%)				
Hispanic	13,185 (24.7)	2,210 (22.2)	10,975 (25.2)	***<0.001***
Non-Hispanic	39,363 (73.7)	7,503 (75.5)	31,860 (73.3)	
Unknown[b]	864 (1.6)	224 (2.3)	640 (1.5)	
Region, n (%)				
Northeast	5,017 (9.4)	1,339 (13.5)	3,678 (8.5)	***<0.001***
South	18,588 (34.8)	3,405 (34.3)	15,183 (34.9)	
Midwest	7,475 (14.0)	1713 (17.2)	5,762 (13.3)	
West	21,795 (40.8)	3,076 (31.0)	18,719 (43.1)	
Unknown	537 (1.0)	404 (4.1)	133 (0.3)	
Marital status, n (%)				
Married	21,103 (39.5)	4,201 (42.3)	16,902 (38.9)	***<0.001***

Single/divorced[c]	29,432 (55.1)	5,155 (51.9)	24,277 (55.8)	
Other[d]	2,877 (5.4)	581 (5.8)	2,296 (5.3)	
Insurance type, n (%)				
Commercial	6,528 (12.2)	1,194 (12.0)	5,334 (12.3)	***<0.001***
Medicaid	10,688 (20.0)	1,217 (12.2)	9,471 (21.8)	
Medicare	26,556 (49.7)	5,367 (54.0)	21,189 (48.7)	
Other government	882 (1.7)	88 (0.9)	794 (1.8)	
Other non-government	445 (0.8)	88 (0.9)	357 (0.8)	
Self-pay	875 (1.6)	126 (1.3)	749 (1.7)	
Unknown	7,438 (13.9)	1,857 (18.7)	5,581 (12.8)	
Nicotine dependence/cigarette use, n (%)	6,610 (12.4)	975 (9.8)	5,635 (13.0)	***<0.001***
History of alcohol abuse/dependence, n (%)	3,253 (6.1)	373 (3.8)	2,880 (6.6)	***<0.001***
History of drug abuse or dependence, n (%)	4,568 (8.6)	540 (5.4)	4,028 (9.3)	***<0.001***
BMI, mean (SD)	**n=48,921**	**n=8,812**	**n=40,109**	***0.034***
	30.2 (8.8)	30.0 (8.3)	30.2 (8.9)	
BMI, n (%)				
Underweight (<18.5)	2,019 (3.8)	302 (3.0)	1,717 (3.9)	***<0.001***
Normal (18.5-25)	12,670 (23.7)	2,269 (22.8)	10,401 (23.9)	
Overweight (25-30)	13,234 (24.8)	2,544 (25.6)	10,690 (24.6)	
Obese (30+)	20,998 (39.3)	3,697 (37.2)	17,301 (39.8)	
Unknown[f]	4,491 (8.4)	1,125 (11.3)	3,366 (7.7)	
Cohort medication use, n (%)				
Patiromer first	434 (0.8)	434 (4.4)	0 (0.0)	***<0.001***
Patiromer only	9,503 (17.8)	9,503 (95.6)	0 (0.0)	
SZC first	451 (0.8)	0 (0.0)	451 (1.0)	
SZC only	43,024 (80.6)	0 (0.0)	43,024 (99.0)	
Comorbidities, n (%)				
Any heart failure	19,365 (36.3)	3,362 (33.8)	16,003 (36.8)	***<0.001***
Acute heart failure	1,115 (2.1)	151 (1.5)	964 (2.2)	***<0.001***
Acute chronic heart failure	8,083 (15.1)	1,387 (14.0)	6,696 (15.4)	***<0.001***
Chronic heart failure	5,402 (10.1)	992 (10.0)	4,410 (10.1)	*0.631*
Unknown heart failure	4,765 (8.9)	832 (8.4)	3,933 (9.0)	***0.034***
Edema	6,577 (12.3)	1,257 (12.6)	5,320 (12.2)	*0.259*
Cardiomyopathy	7,665 (14.4)	1,340 (13.5)	6,325 (14.5)	***0.006***

Ischemic heart disease	12,114 (22.7)	1,866 (18.8)	10,248 (23.6)	***<0.001***
Angina	676 (1.3)	110 (1.1)	566 (1.3)	*0.117*
Myocardial infarction	8,898 (16.7)	1,290 (13.0)	7,608 (17.5)	***<0.001***
Coronary artery procedure	1,160 (2.2)	211 (2.1)	949 (2.2)	*0.714*
Atrial fibrillation	12,446 (23.3)	2,112 (21.8)	10,334 (23.8)	***<0.001***
Cardiac conduction disorder	5,641 (10.6)	963 (9.7)	4,678 (10.8)	***0.002***
Other cardiac dysrhythmia	5,373 (10.1)	781 (7.9)	4,592 (10.6)	***<0.001***
Hyperkalemia	38,077 (71.3)	6,558 (66.0)	31,519 (72.5)	***<0.001***
Hypokalemia	6,899 (12.9)	903 (9.1)	5,996 (13.8)	***<0.001***
Hypermagnesemia	570 (1.1)	88 (0.9)	482 (1.1)	*0.051*
Hypomagnesemia	5,036 (9.4)	813 (8.2)	4,223 (9.7)	***<0.001***
Hypercalcemia	1,719 (3.2)	252 (2.5)	1,467 (3.4)	***<0.001***
Hypocalcemia	3,389 (6.3)	476 (4.8)	2,913 (6.7)	***<0.001***
Hypernatremia	3,547 (6.6)	404 (4.1)	3,143 (7.2)	***<0.001***
Hyponatremia	14,650 (27.4)	2,047 (20.6)	12,603 (29.0)	***<0.001***
CKD (stage 1-2)	1,629 (3.0)	310 (3.1)	1,319 (3.0)	*0.654*
CKD (stage 3-5)	25,502 (47.7)	4,932 (49.6)	20,570 (47.3)	***<0.001***
Proteinuria	2,517 (4.7)	620 (6.2)	1,897 (4.4)	***<0.001***
Hypertension	44,077 (82.5)	8,029 (80.8)	36,048 (82.9)	***<0.001***
Hyperlipidemia	28,000 (52.4)	5,214 (52.5)	22,786 (52.4)	*0.916*
Type I diabetes	1,877 (3.5)	428 (4.3)	1,449 (3.3)	***<0.001***
Type II diabetes	30,867 (57.8)	5,741 (57.8)	25,126 (57.8)	*0.971*
Diabetic nephropathy	20,551 (38.5)	4,197 (42.2)	16,354 (37.6)	***<0.001***
Diabetic retinopathy	3,496 (6.5)	793 (8.0)	2,703 (6.2)	***<0.001***
Diabetic neuropathy	8,713 (16.3)	1,836 (18.5)	6,877 (15.8)	***<0.001***
Diabetes with peripheral circulation disorder	5,249 (9.8)	1,017 (10.2)	4,232 (9.7)	*0.131*
Diabetes without complications	22,565 (42.2)	4,111 (41.4)	18,454 (42.4)	*0.050*
Peripheral vascular disease	10,060 (18.8)	1,911 (19.2)	8,149 (18.7)	*0.263*
Ischemic stroke	2,920 (5.5)	506 (5.1)	2,414 (5.6)	*0.068*
Transient ischemic attack	762 (1.4)	162 (1.6)	600 (1.4)	*0.058*
COPD	10,785 (20.2)	1,778 (17.9)	9,007 (20.7)	***<0.001***
Asthma	3,685 (6.9)	583 (5.9)	3,102 (7.1)	***<0.001***

Obstructive sleep apnea	7,818 (14.6)	1,543 (15.5)	6,275 (14.4)	***0.005***
Liver disease	10,506 (19.7)	1,472 (14.8)	9,034 (20.8)	***<0.001***
Hyperthyroidism	679 (1.3)	121 (1.2)	558 (1.3)	*0.597*
Hypothyroidism	8,659 (16.2)	1,594 (16.0)	7,065 (16.3)	*0.609*
Osteoporosis	2,045 (3.8)	360 (3.6)	1,685 (3.9)	*0.236*
Depression	7,708 (14.4)	1,490 (15.0)	6,218 (14.3)	*0.077*
Dementia	3,225 (6.0)	540 (5.4)	2,685 (6.2)	***0.005***
Anxiety	8,339 (15.6)	1,376 (13.8)	6,963 (16.0)	***<0.001***
Falls	8,507 (15.9)	1,376 (13.8)	7,131 (16.4)	***<0.001***
Concomitant medications, n (%)				
ACE inhibitor (any medication that includes an ACE inhibitor, including combinations)	15,183 (28.4)	2,655 (26.7)	12,528 (28.8)	***<0.001***
ARBs (any medication that includes an ARB, including combinations)	9,418 (17.6)	1,751 (17.6)	7,667 (17.6)	*0.973*
Sacubitril/valsartan	1,326 (2.5)	276 (2.8)	1,050 (2.4)	***0.036***
MRA (any medication that includes an MRA, including combinations)	6,259 (11.7)	978 (9.8)	5,281 (12.1)	***<0.001***
Any beta-blockers (any medication that includes beta-blockers, including combinations)	34,954 (65.4)	6,593 (66.3)	28,361 (65.2)	***0.035***
Carvedilol	12,585 (23.6)	2,608 (26.2)	9,977 (22.9)	***<0.001***
Metoprolol	19,354 (36.2)	3,457 (34.8)	15,897 (36.6)	***<0.001***
Bisoprolol	243 (0.5)	46 (0.5)	197 (0.5)	*0.896*
Other	14,525 (27.2)	2,453 (24.7)	12,072 (27.8)	***<0.001***
CCBs (any medication that includes CCBs, including combinations)	25,808 (48.3)	4,986 (50.2)	20,822 (47.9)	***<0.001***
Loop diuretics (any medication that includes a loop diuretic, including combinations)	30,726 (57.5)	5,497 (55.3)	25,229 (58.0)	***<0.001***
Thiazide diuretics (any medication that includes a thiazide diuretic, including combinations)	6,638 (12.4)	1,192 (12.0)	5,446 (12.5)	*0.148*
SGLT-2 inhibitors (any medication that includes an SGLT-2 inhibitor, including combinations)	1,235 (2.3)	167 (1.7)	1,068 (2.5)	***<0.001***
SPS	10,009 (18.7)	2,915 (29.3)	7,094 (16.3)	***<0.001***
NSAIDs	30,977 (58.0)	5,450 (54.8)	25,527 (58.7)	***<0.001***

ACE, angiotensin-converting enzyme; ARB, angiotensin receptor blockers; BMI, body mass index; CCB, calcium channel blockers; CKD, chronic kidney disease; COPD, chronic obstructive pulmonary disease; MI, myocardial infarction; MRA, mineralocorticoid receptor antagonists; NSAID, nonsteroidal anti-inflammatory drug; SD, standard deviation; SGLT, sodium-glucose co-transporter; SPS, sodium polystyrene sulfonate; SZC, sodium zirconium cyclosilicate.

[a]Null, other, and multiple reported.

[b]Null and multiple reported.
[c]Single, widowed, divorced, never married, and unmarried.
[d]Domestic partner, separated, legally separated, other, polygamous, multiple reported, and null.
[e]Commercial, Medicare, Medicaid, other government, and other non-government.
[f]Values less than 10 and greater than 100 were recoded as unknown.
NOTE: P-value >0.05 indicates no significant differences between groups.
